# Melanocytes as the source of the increased melanisation in pigmented epithelial tumours: a holistic approach

**DOI:** 10.55730/1300-0144.5362

**Published:** 2022-01-15

**Authors:** Asuman KİLİTCİ, Ömer Faruk ELMAS, Necmettin AKDENİZ, Mehmet GAMSIZKAN

**Affiliations:** 1 Department of Pathology, Faculty of Medicine, Kırşehir Ahi Evran University, Kırşehir, Turkey; 2 Department of Dermatology, Faculty of Medicine, Kırıkkale University, Kırıkkale, Turkey; 3 Department of Dermatology, Memorial Şişli Hospital, İstanbul, Turkey; 4 Department of Pathology, Faculty of Medicine, Düzce University, Düzce, Turkey

**Keywords:** Pigmentation, melanisation, basal cell carcinoma, seborrheic keratosis, SOX10

## Abstract

**Background/aim:**

We aimed to elucidate the causes of the increased melanisation in basal cell carcinoma (BCC) and seborrheic keratosis (SK), and the role of melanocytes in this process.

**Materials and methods:**

This study was a retrospective-cohort study conducted in the pathology department of a university hospital between January 2019 and October 2020. Forty-nine SK and 30 pigmented BCC were included in our study. SRY-box transcription factor 10 (SOX10), CD68, and Masson–Fontana staining was used for analysis in all samples. A representative section of each specimen was photographed under ×400 magnification to facilitate the assessments of the morphology of the melanocytes and their following morphometric parameters: density, nuclear diameter, and distribution. The density of pigmented keratinocytes in the lesional epidermis was scored. The nuclear diameters of melanocytes located in the nonlesional epidermis, the density of the melanophages, and the presence or absence of ulceration and solar elastosis were also recorded.

**Results:**

The morphometric findings confirmed a statistically significant increase in melanocyte density in the BCC group compared with that in the SK group (p < 0.001). Moreover, the nuclear minor diameters in the melanocytes of the BCC sections were significantly higher than those in the SK specimens (p < 0.001). The epidermal melanocytes were distributed diffusely in almost all BCC specimens (96.7%), whereas they were mainly limited to the basal layer in the majority of the SK sections (59.2%).

The number of epidermal melanised keratinocytes with a score of 3 was significantly higher in the SK group (n = 31; 63.2%) than in the BCC group (n = 6; 20%) (p = 0.001), and they were the main cells representing the pigmented appearance of the tumours. No significant difference was found between both tumour groups in terms of their melanophage density scores (p = 0.206).

**Conclusion:**

This study is the first step towards an objective quantification of the melanocytes in pigmented epithelial tumours and may provide a morphological background for future studies on these skin lesions.

## 1. Introduction

Melanocytes, which are melanin-producing cells that originate from the neural crest, are located in the basal cell layer of the epidermis. These cells produce melanin from l-3,4-dihydroxyphenylalanine (l-DOPA) after synthesising the latter from tyrosine. Aside from protecting the skin from ultraviolet radiation through their production of melanin, melanocytes also play a role in the immune system [1]. However, melanin synthesis has also been found to occur in basal cell carcinoma (BCC) and seborrheic keratosis (SK), two common pigmented tumours of the skin epithelium. Despite that increased melanin production is common in benign and malignant epithelial skin tumours, only a limited number of studies have focused on the exact cause of the increased melanisation and the role played by melanocytes in the pathogenesis of these neoplasms. It has been reported that endothelin-1, a keratinocyte-derived cytokine with a stimulatory effect on melanocytes, may play a role in the melanisation of SK [2].

Melanin pigments, which are synthesised and deposited in melanocyte organelles called melanosomes, are generally transferred to an average of 36 keratinocytes through the latter’s phagocytosis of the melanosome-loaded dendritic ends of the melanocytes. Protease-activated receptor 2 (PAR-2), a key receptor expressed on keratinocytes, is involved in melanosome transfer [3]. Other important regulators of this process are kinesin and actin-related myosin V, the latter of which protects the peripheral melanosomes and prepares them for transfer to the surrounding keratinocytes [4]. It is well known that any inflammatory process involving melanin transfer in the basal layer of the epidermis, specific enzyme defects, and the destruction of melanocytes are causes of hypopigmentation [1]. However, the pathogenesis of hyperpigmentation is not well understood.

Currently, there are many markers in use for the histopathological identification of melanocytes. SRY-box transcription factor 10 (SOX10), a transcription factor that plays a role in the development of melanocytes, is considered a suitable nuclear marker for the morphometric analysis of these cells because of its high sensitivity and specificity [5–7]. In this context, we surmised that it would be easy to identify and evaluate the melanocytic cells in BCC and SK using this marker.

In this study, we aimed to elucidate the causes of the increased melanisation in BCC and SK, and the role of melanocytes in this process, by investigating the morphological and morphometric differences in SOX10-identified melanocytes in these two types of skin tumours. We also aimed to assess the contribution of melanophages in other causes of increased melanisation by using the CD68 antibody.

## 2. Materials and methods

### 2.1. Patients

This study was a retrospective-cohort study conducted in the pathology department of a university hospital between January 2019 and October 2020. Histopathological slides of skin tumour tissue from patients who were previously diagnosed with BCC or SK were retrieved from the pathology files and reexamined. Patients whose diagnoses were confirmed by at least two pathologists (A.K.; M.G.) experienced in dermatopathology were included in the study, and their clinical and demographic characteristics were recorded along with the histopathological subtypes of the tumours.

### 2.2. Inclusion and exclusion criteria

The inclusion criteria were as follows: a precise histopathological diagnosis of BCC or SK; availability of sufficient clinical information; sufficient pathology specimens for analysis; pathologically confirmed clear surgical margins; and the presence of pigmentation in at least 1% of the whole section. Collision tumours were excluded.

### 2.3. Histopathological and immunohistochemical analysis

**Figure 1:**
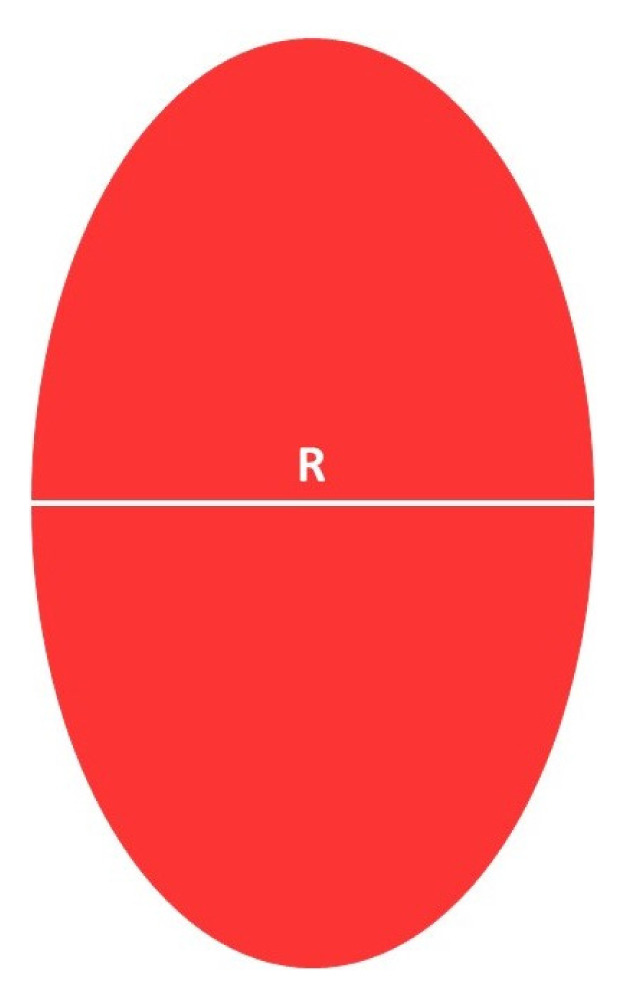
A schematic presentation of nuclear minor diameter (R) of a melanocyte.

**Figure 2:**
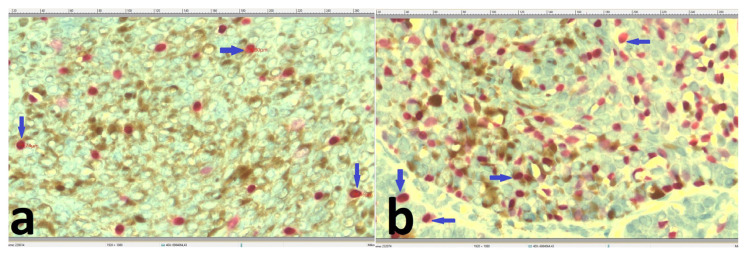
Melanocyte density was lower in the SK (×400) (a), compared to the BCC (×400) (b) (blue arrows indicate some SOX-10 positive melanocytes).

**Figure 3:**
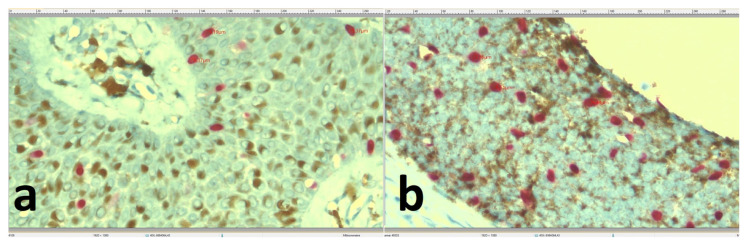
Measuring the short axis of the three largest melanocytes. The nuclear minor diameters in the melanocytes of the SK sections (×400) (a) were lower than those in the BCC specimens (×400) (b).

**Figure 4:**
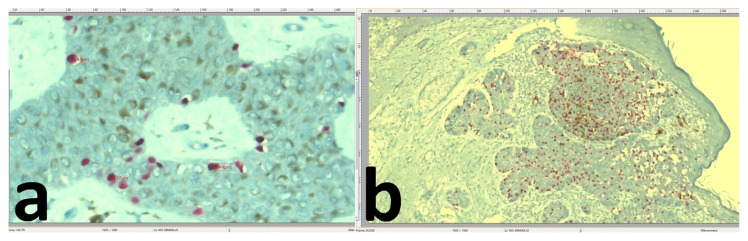
The tendency of the SOX10-positive melanocytes to show diffuse/epidermal basal distribution. Melanocytes in SK were not diffuse in the tumour lobules but mostly spread along the basal layer of the tumour (x400) (a), in the pigmented areas of the BCC, melanocytes were interspersed not only along the basal layer of the tumour but also among the tumour cells in the central part of the tumour nest (×100) (b).

**Figure 5:**
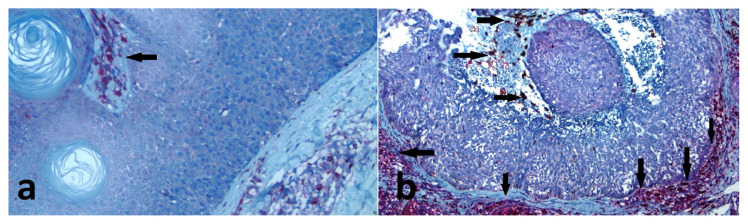
The melanophages were found in the connective tissue surrounding the tumour masses (arrows). Melanophages detected in the dermis and cystic spaces using CD68 staining were found to occur less frequently in the SK (×200) (a) than in the BCC (×100) (b).

Representative paraffin-embedded blocks of tissue with the most intense pigmentation in the epidermis were sectioned and the tissue slices were processed for immunohistochemical examination using the standard avidin-biotin-peroxidase complex method. The antibody used was raised against SOX10 (EP268, BioSB, Santa Barbara, CA, USA), and a red chromogen was applied for antigen localisation. Proper positive and negative controls were run in parallel. Cells with a nuclear staining pattern indicated positive immunostaining for SOX10, whereas those with no staining or a cytoplasmic staining pattern indicated negative immunostaining. A representative section of each specimen was photographed under ×400 magnification to facilitate the assessments of the morphology of the melanocytes and their following morphometric parameters: density, nuclear diameter, and distribution (Figure 1–4). For the melanocyte density, the hot spot field of SOX10 positive melanocytes in each tissue section was determined and it was counted in high power of magnification (×400). (Figure 2). The nuclear diameter was obtained by measuring the short axis of the three largest melanocytes within a 500 μm (×400) segment of the section and then recording the mode (Figure 3). The tendency of the SOX10-positive melanocytes to show diffuse or epidermal basal distribution was noted (Figure 4). The nuclear diameters of melanocytes located in the nonlesional epidermis, the density of the melanophages, and the presence or absence of ulceration and solar elastosis were also recorded. The density of melanophages in the dermis adjacent to the tumour was graded using CD68 (KP1, MS-397-P; Thermo Scientific; Fremont, CA, USA) and Masson–Fontana staining (0–5:0, 6–10:1, 11–15:2, >15:3) (Figure 5). To calculate the melanophage density, on each slide, a representative area was chosen. The number of melanophages in one high-power (×40 objective) field was counted. The possibility of siderophage formation was excluded using Prussian blue staining. The density of pigmented keratinocytes in the lesional epidermis was scored semiquantitatively (0: absent; 1: mild/focal; 2: moderate; 3: dense/diffuse).

### 2.4. Statistical analysis

Numerical data were summarized with mean±standard deviation or median (min-max) as appropriate, and categorical data were summarized with frequencies and percentages. The normality assumption of the data was evaluated with the Shapiro-Wilk test. Independent samples t-test or Mann–Whitney U test were used to compare the data between the two groups depending on data distribution in each measurement. One-way analysis of variance (ANOVA) was also used to compare data for multiple groups (tumor site: head & neck, trunk, extremities). Pearson’s χ2 test or Fisher-Freeman-Halton test was used to analyze categorical data according to the expected count rule. Statistical analyses were done using SPSSv.22 package (SPSS IBM Statistics, Armonk, New York, USA), and the level of significance was considered as 0.05.

### 2.5. Ethics approval

All the procedures followed the Helsinki Declaration and the study was approved by the local Institutional Review Board (Decision number: 2020-09/62).

## 3. Results

In total, 49 patients with SK (32 males, 17 females) and 30 patients with BCC (19 males, 11 females) were included in the study. The mean age of the patients was 57.2 years (range: 17–80 years) in the SK group and 67.4 years (range: 42–87 years) in the BCC group (p = 0.01) (Table).

The most common location of the BCC was the head–neck region (n = 26; 86.6%), followed by the trunk (n = 2; 6.7%) and extremities (n = 2; 6.7%). The most common histological subtype of BCC was the solid type (n = 9; 30%), followed by the micronoduler (n = 8; 26.7%), nodulocystic (n = 6; 19.9%), adenoid (n = 3; 10%), superficial (n = 2; 6.7%), and infiltrative (n = 2; 6.7%) types.

The most common location of the SK was the head–neck region (n = 24; 49%), followed by the trunk (n = 18; 36.7%) and extremities (n = 7; 14.3%). The most common histological subtype was the acanthotic type (n = 29; 59.2%), followed by the hyperkeratotic (n = 9; 18.4%), reticulated (n = 6; 12.2%), and irritated (n = 5; 10.2%) types.

In the SK group, the average number of melanocytes per 100 basal-layer cells was 15.3 and 9.5 for the lesional and nonlesional areas, respectively. Therefore, we found that the melanocyte density in the SK specimens was approximately 1.5 times higher than that in the peritumoural normal controls. There were no statistically significant differences between the males and females in terms of the average number of melanocytes per 100 basal layer cells both in intralesional and extralesional epidermis in SK (p = 0.474, p = 0.231). These measurements could not be made for the BCC group, as the melanocytes are widely distributed in this type of skin cancer as opposed to that observed in SK.

Morphometrically, melanocyte density was lower in the SK group (Figure 2a) compared to BCC group (Figure 2b). This finding was statistically significant (p < 0.001). Moreover, the nuclear minor diameters in the melanocytes of the SK sections (Figure 3a) were significantly lower than those in the BCC specimens (p < 0.001) (Figure 3b) (Table).

The mean diameters of peritumoural melanocytes were not significantly different between the BCC and SK groups (p = 0.821) (Table). Although the melanocytes appeared oval–round in shape in both types of tumours, they were relatively more uniform in size and shape in the SKs. The epidermal melanocytes were mainly limited to the basal layer in the majority of the SK sections (Figure 4a) (59.2%), whereas they were distributed diffusely in almost all BCC specimens (96.7%) (Figure 4b).

The number of epidermal pigmented keratinocytes with a score of 3 was significantly higher in the SK group (n = 31; 63.2%) than in the BCC group (n = 6; 20%) (p = 0.001), and they were the main cells representing the pigmented appearance of the tumours (Table). Melanophages detected in the dermis and cystic spaces using CD68, Masson–Fontana, and Prussian blue staining were found to occur more frequently in the BCC specimens (90%) than in the SK sections (71.4%) (Figure 5a,5b). However, no significant difference was found between both tumour groups in terms of their melanophage density scores (p = 0.206) (Table). The melanophages were found in the connective tissue surrounding the tumour masses.

Solar elastosis occurred more frequently in the BCC group (n = 23; 76.6%) than in the SK group (n = 16; 32.6%) (p = 0.000), and its presence was significantly associated with increasing age (p = 0.010) and elevated melanocyte density (p = 0.002). We found the mean nuclear minor diameter for each case and compared it to solar elastosis. There was a slight increase in melanocyte diameter based on solar elastosis (5.85 μm vs. 5.57 μm). However, there was no statistically significant difference for the parameter of melanocyte diameter according to the presence or absence of solar elastosis (p > 0.05) among the whole study population. Although there was a slight increase in melanocyte diameter in the presence of solar elastosis, no statistical significance was found in both the BCC (mean melanocyte diameter: 6.21 μm vs. 6.14 μm; p > 0.05) and SK groups (mean melanocyte diameter: 5.45 μm vs. 5.27 μm; p > 0.05).

In total, 13 BCC specimens (43.3%) and 1 SK section (2%) showed ulceration (p < 0.0001) (Table). There were no significant differences between ulcerated and nonulcerated cases for the parameters of melanocyte diameter, melanocyte density, pigmented keratinocyte density, and dermal melanophages. Moreover, the presence of ulcers in patients with BCC did not differ significantly according to sex (p = 0.132).

## 4. Discussion

Nowadays, in addition to traditional histopathology for the diagnosis and surgical treatment of skin cancers, the quantitative assessment of melanocytic parameters using image analysis is becoming increasingly important as well. To investigate whether there are significant differences in the morphometry of melanocytes between pigmented BCC and SK, SOX10 staining of archived formalin-fixed paraffin-embedded tissue sections was carried out in this study. The nuclear staining quality of SOX10 facilitates localisation of the melanocytes within the epidermis and measurement of the nuclear parameters by image analysis [5,6].

Melanin is usually formed in nodular, micronodular, multifocal superficial, or follicular variants of BCC [8]. However, in our study, the most common tumour subtypes found to contain pigmentation were nodular, micronodular, and nodulocystic, and these were considered to fall into the category of pigmented BCC based on WHO 2018 classification.

The melanocytes in SK were mostly basally located, and their nuclear sizes were significantly smaller than those of the melanocytes in BCC, with a ratio of approximately 1.16:1. The melanocytes in SK appeared to be associated with the inactive state of the cell as opposed to the hyperactive, large melanocytes in BCC.

The origin of melanocytes in pigmented BCC is unclear, albeit different hypotheses have been proposed. For example, the melanocytes may have migrated from the neighbouring epidermis. Given that several previous studies have reported the presence of melanocyte-attracting chemotactic factors in some tumours, including BCC, the possibility of nonneoplastic epidermal melanocytes migrating to and colonising tumour areas after BCC formation is quite feasible [9–11].

It is widely accepted that there is a close relationship between the derivation of BCC and the histogenesis of hair follicles. Melanocytes may also originate from BCC cells in hair follicles. These findings have provided evidence of a correlation between melanocytes in pigmented BCC and those in the hair follicle. In other words, melanocytes in pigmented BCC may be derived from hair follicles accompanied by BCC cell differentiation [9–11]. Taken together, these reports and the current findings provide a possible explanation (at least in part) for the observed racial trends in pigmented BCC prevalence; that is, clinically pigmented BCC occurs more commonly in races with naturally black hair than in those with naturally blonde hair [12]. Considering that SK also results from basaloid keratinocytes that had matured from basal cells, it may explain the increase in melanised keratinocytes and melanocytes in this type of skin tumour as well.

In the normal human epidermis, melanocytes are in close contact with a large number of keratinocytes, supplying them with melanin [13,14]. Keratinocytes are active secretory cells that stimulate melanocyte growth in a regulated manner in vitro [15]. Benign melanocytes in BCC were observed to have diverged when in the normal human epidermis, suggesting that neoplastic keratinocytes may still regulate the growth of the melanocytes that harbour them [16]. This study also found that the number of malignant melanocytes in pure melanoma in situ (MIS) were lower than malignant melanocytes in a BCC tumour in collision with melanoma in situ (BCC+MIS) [16]. It is also possible that a BCC is easily populated by melanomas because the cells are met with little resistance by the poorly adherent BCC cells [16]. Although the keratinocyte-melanocyte interaction unit may still work in benign epithelial tumours such as SK, it can be assumed that the unit is disrupted as it shifts to the malignant epithelial tumour or collision tumour spectrum, suggesting that different mechanisms or regulators that stimulate the proliferation of melanocytes cannot effectively limit their proliferation as well.

Florell et al. hypothesised that the BCC entraps melanocytes as the tumour grows, given that melanocytes are located in the epidermal basal layer from which BCCs arise [16]. They argued that a similar phenomenon was observed in studies of pigmented epidermotropic breast cancer metastasis in which melanocytes were colonised [17–20]. However, in our study, melanocytes were seen throughout the basal layer in the majority of SKs. Considering that SKs also originate from basal cells, our results do not support the view of Florell et al. [16]. At this point, we can only speculate on whether these melanocytes originated from the basal cells. It is possible that pluripotent stem cells differentiated into melanocytes. In this context, electron microscopy studies are needed to evaluate the ultrastructures of the melanocytes in basal cells and in ordinary melanocytic lesions.

It has been reported that the pigmented areas of SK and BCC are rich in dendritic melanocytes, which have been suggested to provide melanosome transfer between two tumour cells [16,18,20–22]. However, it is notable in our study that the melanocytes in the pigmented parts of both tumours were oval-round in shape rather than dendritic, which again raises the question of whether the melanocytes had originated from basal cells rather than the neural crest.

Our morphometric and immunohistochemical findings showed that in the pigmented areas of the BCC, melanocytes were interspersed not only along with the basal layer of the tumour but also among the tumour cells in the central part of the tumour nest, which is consistent with the findings of Lao et al. [12]. However, using the dihydroxyphenylalanine (DOPA) reaction, Deppe et al. demonstrated that melanocytes were localised predominantly in the periphery of the BCC [23]. However, contrary to the results reported in the literature, we found that the melanocytes in SK were not diffuse in the tumour lobules but mostly spread along with the basal layer of the tumour [24]. Moreover, they were distributed in a ratio of approximately 15 to every 100 basal cells along the basal lamina. The melanocyte density in the SK specimens was approximately 1.5 times higher than that in the peritumoural normal controls.

It has been reported that pigmented BCCs contain more melanocytes than their nonpigmented counterparts and that the clinically pigmented appearance is provided by the melanocytes rather than the melanin pigments [25]. According to our present findings, we can state that the melanocytes and melanophages contributed more to the clinically pigmented appearance in BCCs than they did in SKs. However, the source of the darkly pigmented appearance of both tumours was melanin-containing keratinocytes, being more predominantly so for the SK specimens.

It has been suggested that ulceration in pigment-containing BCCs causes melanin pigments to be released and subsequently phagocytosed by the surrounding tumour cells [22]. However, in our study, no significant relationship could be established between the presence of ulceration and the proliferation of melanocytes or the density of epidermal pigmented keratinocytes.

Another finding in our study was that chronic sun damage increased the density of melanocytes in the lesions. Although there was no statistically significant difference in the diameters of melanocytes between both tumour groups, we believe that chronic sun damage may affect both the density and diameter. The evaluation of a larger case series is needed to reach a definitive conclusion on this aspect.

## 5. Conclusion

In this study, differences in the morphology and morphometry of melanocytes were found between those in BCC and those in SK tissue specimens. However, as discussed above, because of the possibility of pluripotent stem cells differentiating into melanocytes, the determination of the melanocyte source in pigmented epithelial tumours on the basis of a morphometric analysis alone is controversial. In this context, an electron microscopy study is needed to evaluate the ultrastructural features of the melanocytes in ordinary melanocytic lesions and in basal cells. The limitation of our study is the absence of such ultrastructural findings and a partially small case population. Notwithstanding, this study is the first step towards an objective quantification of the melanocytes in pigmented epithelial tumours and may provide a morphological background for future studies on these skin lesions.

## Figures and Tables

**Table:** Clinicopathological characteristics of the study population.

		SK (n = 49)	BCC (n = 30)	p value
**Gender**				
	*Male*	*32*	*19*	0.859
	*Female*	*17*	*11*	
**Age (17–87 years)**		*57.3*	*67.4*	0.01
**Tumor site**				
	*Head/neck*	*24*	*26*	0.003
	*Trunk*	*18*	*2*	
	*Extremities*	*7*	*2*	
**Solar elastosis**				
	*Yes*	*16*	*23*	<0.001
	*No*	*33*	*7*	
**Melanocytic distribution**				
	*Diffuse*	*20*	*29*	<0.001
	*Peripheral*	*29*	*1*	
**Melanocyte density in the epithelium (per 500 μm;** ×**400)**		*23.5*	*63.5*	<0.001
**Normal melanocytic nuclear diameter (μm)**		*5.3*	*5.2*	0.821
**Intralesional melanocytic diameter (μm)**		*5.34*	*6.19*	<0.001
**Epidermal pigmented keratinocyte density**				
	*1*	*5*	*9*	0.001
	*2*	*13*	*15*	
	*3*	*31*	*6*	
**Dermal melanophages**				
	*0*	*14*	*3*	0.206
	*1*	*18*	*11*	
	*2*	*9*	*9*	
	*3*	*8*	*7*	
**Epidermal ulceration**				
	*Yes*	*1*	*13*	<0.001
	*No*	*48*	*17*	
